# American Medical Association

**Published:** 1875-04

**Authors:** W. B. Atkinson

**Affiliations:** Permanent Secretary


### Editorial.

We have received the following from the Secretary of the American Medical Association to which we would direct the attention of our readers:

American Medical Association.—The Twenty-sixth Annual Session will be held in the city of Louisville, Ky., on Tuesday, May 4, 1875, at 11 A. M.

"The delegates shall receive their appointment from permanently organized State Medical Societies, and such County and District Medical Societies as are recognized by representation in their respective State Societies, and from the Medical Department of the Army and Navy of the United States."

"Each State, County and District Medical Society entitled to representation shall have the privilege of sending to the Association one delegate for every ten of its regular resident members, and one for every additional fraction of more than half that number; provided, however, that the number of delegates for any particular State, territory, county, city, or town shall not exceed the ratio of one in ten of the resident physicians who may have signed the Code of Ethics of the Association."

"The Chairmen of the several sections shall prepare and read in the general session of the Association, papers on the advances and discoveries of the past year in the branches of science included in their respective sections. * * *"—By-Laws, Article 2, Section 4.

The following amendments to the Plan of Organization are to be acted upon :

By Dr. H. B. Baker, Michigan—" The officers of the several Sections shall be nominated by the Section in and for which said officers are to serve"

By Dr. Adams Jewett, Ohio—" The permanent members shall consist of all those who have served in the capacity of delegate, and of such other members as shall have received the appointment by unanimous vote, and of all others who, being members in good standing of any State or local Medical Society entitled to representation in this Body, shall, after being vouched for by at least three members, be elected to membership by a vote of three-fourths of the delegates in attendance, and shall continue such so long as they remain in good standing in the Body of which they were members when elected to membership in this Association, and comply with the requirements of its by-laws."

Secretaries of all State Medical Societies that have adopted the Code of Ethics are respectfully requested to forward to the undersigned a complete list of officers, with their post-office addresses, of those County and District Medical Societies entitled to representation in their respective bodies. This is the only guide for the Committee of Arrangements in determining as to the reception of delegates. It will also enable the Permanent Secretary to present a correct report of Medical organizations in fellowship with the Association.

W. B. ATKINSON, M. D., Permanent Secretary.

